# A medical calculator to determine testicular volumes matching ultrasound values from the width of the testis obtained in the scrotum with a centimeter ruler

**DOI:** 10.1186/s13633-017-0053-y

**Published:** 2017-11-21

**Authors:** Juan F. Sotos, Naomi J. Tokar

**Affiliations:** 1 Department of Pediatric, College of Medicine, The Ohio State University, Nationwide Children’s Hospital, Section of Pediatric Endocrinology, 700 Children’s Drive, Columbus, Ohio 43205 USA; 2 Nationwide Children’s Hospital, Section of Pediatric Endocrinology, 700 Children’s Drive, Columbus, Ohio 43205 USA

**Keywords:** Testicular volumes, Genital development in males, Genital stages, Orchidometers, Medical calculator

## Abstract

The determination of the testicular volume is of considerable importance to assess the onset, progression and disorders of puberty, abnormal testicular development, and a number of other conditions; and in adults, assessment of fertility. A number of clinical methods have been used for the measurement of testicular volumes in the scrotum: a centimeter ruler, sliding calipers, and orchidometers. All the clinical methods calculate the volumes by the ellipsoid equation, grossly overestimate ultrasound (US) volumes by 70 to 80% for adults, to 150 to 250% for prepubertal subjects, mainly because the inclusion of the scrotal skin and epididymis and may not be accurate of reproducible. Ultrasound measurements have a high degree of accuracy and reproducibility and are the standard for quantitation of testicular volume. Formulas, equivalent to the ellipsoid equations used, were developed to match ultrasound volumes, with corrections of the width and length of the testis obtained in the scrotum, to avoid the inclusion of the scrotal skin (ss) and epididymis.

**A calculator** was developed, requiring only the identification of ① the width of the testis in cm obtained in the scrotum with a ruler (without corrections) (i.e. 0.9, 1.5, 2.0, 2.4 cm etc.) and ② the stage of genital development. The calculator will subtract the scrotal skin for the stage of genital development, from the measurement of the width provided, apply the formula and identify the testicular volume of the subject that matches the US volume.

The calculator will also provide, in a Table form, the values for the different stages of genital development.

**Benefit**: The information provided by the calculator will solve the problem of overestimation by the orchidometers and the external measurements, problems with the reference of values to age, and Tanner stages, would permit assessment of the beginning and progression of puberty, of micro and macroorchidism, and other conditions mentioned.

The determination of the testicular volume is of considerable importance to assess the onset, progression and disorders of puberty, the effect of cryptorchidism and orchiopexy, abnormal testicular development, detection of Klinefelter syndrome, microorchidism or macroorchidism, and a number of other conditions; and in adults, assessment of fertility. Low testicular volume correlates with tubular size, function and spermatogenesis [1].

A number of clinical methods have been used for the measurement of testicular volumes in the scrotum: a centimeter ruler, sliding calipers, and orchidometers (the Prader and the Takihara, probably, the most frequently used). All the clinical methods calculate the volumes by the ellipsoid eq. Width^2^ x Length x (π)/6 (W^2^ x L × 0.52), grossly overestimate ultrasound (US) volumes by 70 to 80% for adults, 100 to 150% for pubertal, and 150 to 250% for prepubertal subjects, mainly because the inclusion of the scrotal skin and epididymis and may not be accurate of reproducible [1].

Ultrasound measurements have a high degree of accuracy and reproducibility and are the standard for quantitation of testicular volume. The ultrasound measurement, however, is somewhat inconvenient because it requires another procedure and, mainly, it is costly. It does not appear practical or reasonable to use US to assess the onset and progression of puberty or assess some of the other conditions that have been mentioned. The US remains the method of choice for the evaluation of extra testicular (i.e. hydrocele, spermatocele, epididymal cyst, varicocele) or intratesticular (i.e. tumors) abnormalities.

The volumes obtained by ultrasound have been calculated by different ellipsoid equations. Some have used only the width (W) and length (L) of the testes, W^2^ x L x $\frac{\uppi\;}{6}$ that when resolved is W^2^x L × 0.52 = Volume. More frequently they have included the height (H), W x H x L × 0.52 and others, recently, have used the constant 0.71 (suggested by Lambert), to closely match the “true” testicular volumes obtained by water displacement, W x H x L × 0.71 = Volume.

Formulas, equivalent to the ellipsoid equations used, with inclusion of the values observed in ultrasound measurements in our hospital, were developed to match ultrasound volumes, with corrections of the width and length of the testis obtained in the scrotum, to avoid the inclusion of the scrotal skin (ss) and epididymis; the Width minus the double scrotal skin (W-ss), to match the US width. With the growth of the testis, the length and width remain proportional, with the length being about 1.5 to 1.57 of the width, with the exclusion of the (ss) and head of epididymis. And the height is usually 0.7 to 0.8 of the width, with the exclusion of scrotal skin and body of epididymis.

For the US eq. W x H x L × 0.52, the equivalent formula would be ((W-ss) x (W-ss) × 0.8 x W-ss) × 1.55 × 0.52 = (W-ss)^3^ × 0.64.

If the constant 0.71 instead of 0.52 is used, then the US equation would be W x H x L × 0.71, and the equivalent formula (W-ss)^3^ × 0.88. These formulas equivalent to the US equations were previously validated and results matching US values of different authors previously reported. (For further details on equations and formulas, see reference [1]).

**The process** to determine testicular volumes matching US values is somewhat simple. The provider measures the width of the testis in the scrotum, by smoothing the scrotal skin around the testis, avoiding compression, and using the ruler. When subtracted by the scrotal skin (ss), this measurement matches or should match the width obtained by US. Then the formula is applied and testicular volumes matching US values obtained.

**A calculator** was developed, so that the provider does not need to make calculations, requiring only the identification of ① the width of the testis in cm obtained in the scrotum with a ruler (without corrections) (i.e. 0.9, 1.5, 2.0, 2.4 cm etc.) and ② the stage of genital development. The calculator will subtract the scrotal skin for the stage of genital development, from the measurement of the width provided, apply the formula and identify the testicular volume of the subject that matches the US volume.

The calculator will also provide, in a Table form (Fig. 1), the values for the different stages of genital development. The volumes provided were calculated by the formula (W-ss)^3^ × 0.88 (equivalent to the equation W x H x L × 0.71): the one preferred presently to obtain the true volume of the testis matching volumes determined by water displacement. If one would like to compare the values obtained by the calculator to those obtained by US in the institution (i.e. W x H x L × 0.52 equivalent to formula (W-ss)^3^ × 0.64), divide the values by 1.365 (0.71/0.52 = 1.365).

**Fig. 1:**
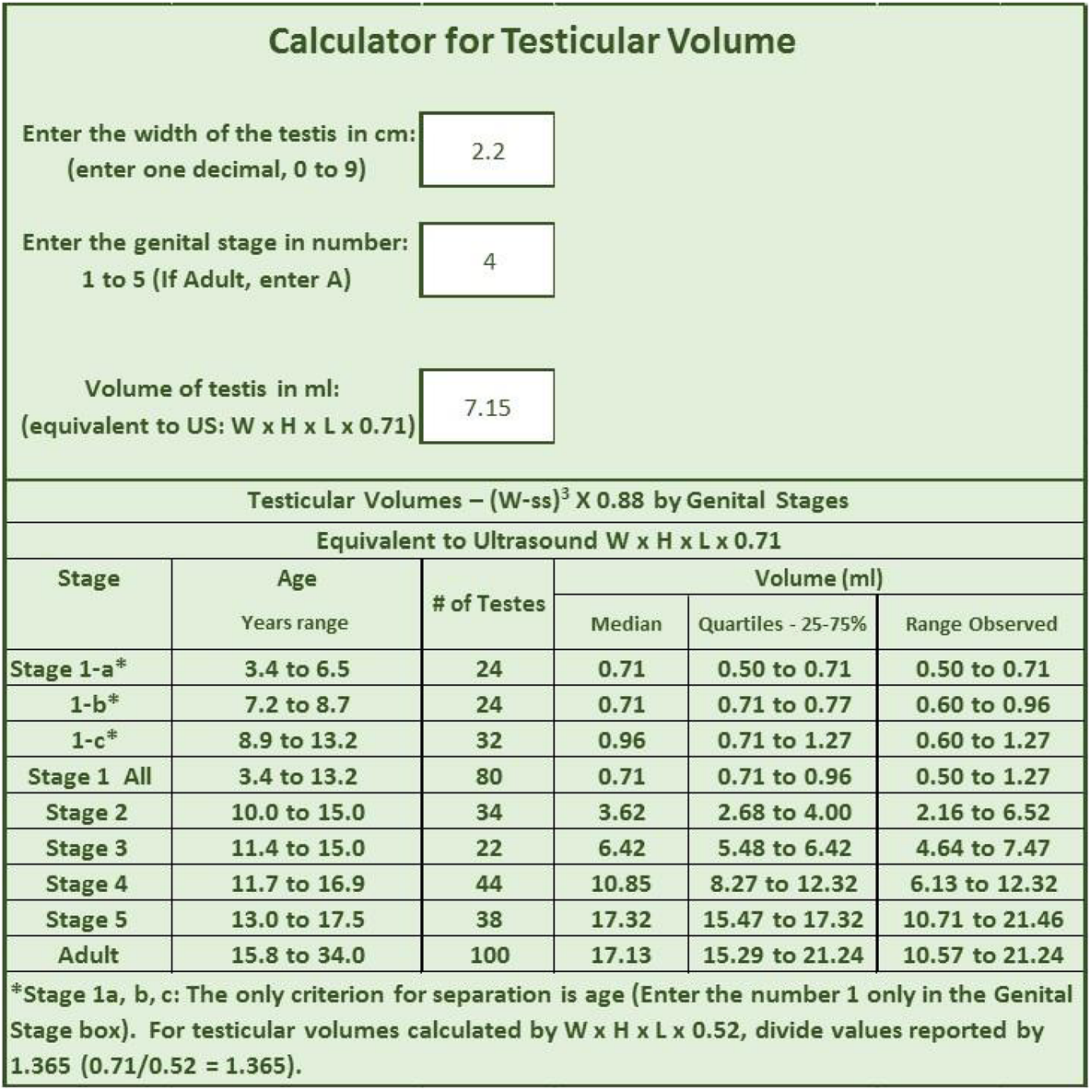
(Picture of the Medical Calculator or Application)

The provider needs to be familiar with the stages of genital development. This may require some explanation to avoid confusion with pubertal stages or Tanner stages, usually used. Pubertal development is determined by 2 events, pubic hair (from adrenarche and gonadarche) and genital development (testis, scrotum and penis from gonadarche). The stages of genital development refer to the changes of the testis, the scrotum, and penis only, separate or independent from pubic hair development. Tanner in 1969 and Marshall & Tanner, in 1970, reported genital development stages of 3 and 4 in males on Tanner stage 1; that is the reason they recommended to analyze the genital development and pubic hair development independently, because one could be out of step with the other.

The main characteristics of the 5 stages of genital development (testes, scrotum, and penis only), separate from pubic hair development, were well defined by Tanner in 1969 by photographs (not shown) and are represented in a sketch in Fig. 2. Our findings and characteristics for the 5 different stages are included in Table 1. One can assess these genital stages visually (Fig. 2) and by the width of the testis in the scrotum and length of the penis (Table 1). These are the stages of genital development described by Tanner and not the Tanner stages or pubertal stages, usually used, that include testicular volumes by orchidometer and pubic hair, without mentioning penis.

**Fig. 2:**
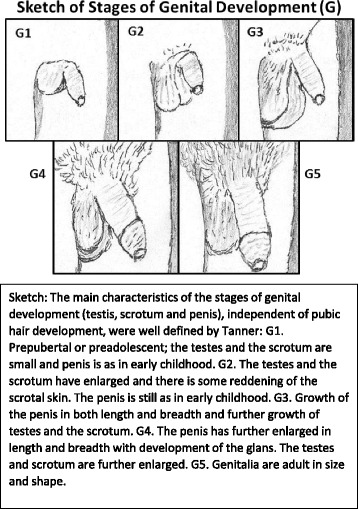
A rough drawing of a photograph published in 1971, for determining stages of genital development (G), (testes, scrotum and penis only), without consideration of pubic hair from Van Wieringen JD, Wafelbakker F, Verbrugge HP, et al. Growth Diagrams 1965 Netherlands: Second National Survey on 0–24 Year Olds. Netherlands Institute for Preventative Medicine TNO. Groningen, The Netherlands: Wolters- Noordhoff; 1971

**Table 1:** Characteristics and Findings for Stages of Genital Development Clinically. Testicular Volumes by (W-ss)^3^ × 0.88^a^

Genital stage	Age range (years)	Testis width in scrotum (cm)	Testicular volume Observed (ml)	Length of penis (cm) Mean ± SD - Observed range
1	Up to 13.25	**> 1.0 to < 1.3**	**< 1.27**	**Early childhood 4.8 ± 0.64 - Observed 3.5 to 6.0**
2	10 to 15	**> 1.5 to 2.1**	**> 2.1 to 6.5**	**Early childhood 5.3 ± 0.75 - Observed 4.0 to 6.5**
3	11.4 to 15	> 1.9 to 2.2	4.6 to 7.4	**Growth of penis 7.4 ± 0.49 - Observed 7.0 to 8.0**
4	11.6 to 17	> 2.1 to 2.6	6 to 12.3	**Development of glans 8.6 ± 1.29 - Observed 6.5 to 12.0 cm**
5	13 to 17.5	**> 2.5 to 3.1**	10.7 to 21.4	9.5 ± 0.99 - Observed 7.5 to 11.0
Adult	> 16	> 2.5 to 3.1	10.6 to 21.2	9.7 ± 1.01 - Observed 8.0 to 11.0

^a^Testicular values obtained by (W-ss)^3^ × 0.88, equivalent to ultrasound values calculated by W x H x L × 0.71. For testicular values calculated by W x H x L × 0.52, divide Values reported by 1.365 (0.71/0.52 = 1.365). The width of the testis was measured. The stage of genital development (testes, scrotum and penis only) as defined by Tanner, and range of years were identified. Testicular volumes were calculated. Penile length was measured. Pubic and other hair observed was recorded, but not included in determining stages 1 to 5 of genital development. [Most helpful findings are bolded]

**Benefit**: The information provided by the calculator will solve the problem of overestimation by the orchidometers and the external measurements, problems with the reference of values to age, and Tanner stages [1], would permit assessment of the beginning and progression of puberty, of micro and macroorchidism, and other conditions mentioned. Assessment of factors affecting differential testicular volumes between the left and right testis (i.e. cryptorchidism, orchyopexy, etc.), could be done by the calculator by the difference of the width obtained in the scrotum. Identification of the stage of genital development is also required, because corrections for the different width of scrotal skin for different stages is needed.

The Application (Calculator) is available to the reader, at no cost, at http://tvcalculator.nchri.org/ and can be accessed from your computer or mobile device.

We certainly encourage the readers to use the calculator and compare the volumes with those obtained on US in their institution, to confirm that volumes matching US volumes can be obtained with a simple measure of the width of the testis in the scrotum. More observations could provide additional values for the range of normal values for different stages of genital development. And, if they have normal distribution, they could be expressed as mean and standard deviations. The additional observations could also provide information on accuracy, and inter-observant difference.

## Abbreviations

H: Height
L: Length
ss: Scrotal skin
US: Ultrasound
W: Width
W-ss: Width minus scrotal skin
π: pi
